# Carotid Intima Media Thickness as a Marker of Atherosclerosis in Ankylosing Spondylitis

**DOI:** 10.1155/2014/839135

**Published:** 2014-04-07

**Authors:** Naveen Gupta, Renu Saigal, Laxmikant Goyal, Abhishek Agrawal, Rajat Bhargava, Arun Agrawal

**Affiliations:** ^1^Department of Medicine, SMS Medical College, Jaipur, India; ^2^Department of Medicine, MG Medical College, Jaipur, India; ^3^Rheumatology Clinic, Mahatma Gandhi Medical College and Hospital, 33 Muktanand Nagar, Gopalpura Byepass, Lane in front of Amarnath Hospital, Jaipur 302018, India

## Abstract

*Aim*. Increased cardiovascular morbidity and mortality have been observed in ankylosing spondylitis because of accelerated atherosclerosis. We measured carotid intima media thickness (CIMT) as a surrogate marker of atherosclerosis in this study. *Methods*. In this study 37 cases of AS and the same number of matched individuals were recruited. CIMT measurements were done using B-mode ultrasound. Disease activity was assessed using Bath ankylosing spondylitis disease activity index (BASDAI), Bath ankylosing spondylitis functional index (BASFI), and Bath ankylosing spondylitis metrological index (BASMI) scores and C-reactive protein (CRP) and erythrocyte sedimentation rate (ESR) levels. *Results*. Mean age of the study groups was 29.43 ± 9.00 years. Average disease duration was 65.62 ± 54.92 months. Twenty-eight (75.68%) of cases were HLA B-27 positive. A significantly increased CIMT was observed in cases as compared to control group (0.62 ± 0.12 versus 0.54 ± 0.04; *P* < 0.001). CIMT in the cases group positively correlated with age (*r* = 0.357; *P* < 0.05), duration of disease (*r* = 0.549; *P* < 0.01), and BASMI (*r* = 0.337; *P* < 0.05) and negatively correlated with ESR (*r* = −0.295; *P* < 0.05). *Conclusions*. Patients of AS had a higher CIMT than those of the control group. CIMT correlated with disease chronicity.

## 1. Introduction

Ankylosing spondylitis (AS) is a chronic inflammatory disease of the axial skeleton, also associated with peripheral arthritis and cardiac, ocular, and gastrointestinal manifestations.

Cardiovascular mortality has been found to be increased in rheumatic diseases, which is attributed to accelerated atherosclerosis [1, 2]. A wide range of cardiovascular features are associated with ankylosing spondylitis, namely, aortitis, aortic regurgitation, and conduction abnormalities [3, 4], along with an increased risk of atherosclerosis related cardiovascular events like myocardial infarction and stroke, leading to a significantly increased mortality [5, 6]. Chronic systemic inflammation is implicated as the driving force behind accelerated atherosclerosis in rheumatic diseases [7, 8].

Among the various screening methods, carotid intima media thickness (CIMT) has gained wide acceptance as a marker of atherosclerosis predicting future cardiovascular events [9, 10].

In this study we have compared CIMT in patients of AS with a matched group of healthy individuals. We also studied the correlation between CIMT and Bath ankylosing spondylitis disease activity index (BASDAI), Bath ankylosing spondylitis functional index (BASFI), and Bath ankylosing spondylitis metrological index (BASMI) scores and C-reactive protein (CRP) and erythrocyte sedimentation rate (ESR) and HLA B-27 status.

## 2. Methods

Thirty-seven patients were recruited from the rheumatology clinic at Sawai Man Singh Hospital, Jaipur, for the study. The same number of healthy individuals matched for age and sex was recruited from the staff of the hospital. Exclusion criteria for both groups were diabetes mellitus, chronic kidney disease, myocardial infarction, hypertension, family history of premature symptomatic coronary heart disease (<55 yrs for males; <65 yrs for females), tobacco chewing, and smoking. Written well-informed consents were obtained from participants of both groups. Necessary clearances were obtained from the institutional ethics committee.

### 2.1. Clinical Assessment

From each participant detailed history was taken and BASDAI, BASFI, and BASMI score were calculated from the questionnaire and physical examination.

### 2.2. Laboratory Evaluation

Fasting venous samples of the patients were collected and evaluated for glucose, creatinine, ESR, CRP, lipid profile, and HLA B-27 status. ESR was measured using Westergren method, C-reactive protein by nephelometry, and lipid profile by colorimetry and the presence of HLA B-27 was tested using polymerase chain reaction (PCR) technique.

### 2.3. CIMT Measurements [10]

CIMT measurements of both study groups were done. All measurements were made by a single examiner. Examination was done in a quiet, cool room (20–25°C). A high resolution B-mode ultrasonography with a 7.5 MHz transducer was used to make the measurements. The subject was placed in supine position with the neck extended and the chin turned contralateral to the side being examined. The distance between the leading edge of the first bright line (the lumen-intima interface) of the far wall and the leading edge of the second bright line (collagen-containing upper layer of tunica adventitia) was taken as the IMT. IMT was measured at three points on the far walls of both the left and the right common carotid arteries (CCA) 10 mm proximal to the carotid bulb. The IMT of these three locations was then averaged to produce the mean IMT for each side. The mean of IMT of each side was taken as the final CIMT.

### 2.4. Statistical Analysis

All measurements were reported as mean ± SD. Chi-square test and Student's* t*-test were used to find the significance among various variables. Correlation between quantitative variables was obtained by calculating Pearson's correlation coefficient. Statistical significance was considered when *P* < 0.05.

## 3. Results

Mean age of the study groups was 29.43 ± 9.00 years. Average disease duration was 65.62 ± 54.92 months. Twenty-eight (75.68%) cases were HLA B-27 positive. We observed a significantly increased CIMT in cases as compared to the control group (0.62 ± 0.12 versus 0.54 ± 0.04; *P* < 0.001). Serum low density lipoprotein cholesterol (LDL-C) was observed to be significantly lower in the cases as compared to control group (80.96 ± 21.66 versus 107.36 ± 25.84 mg/dL; *P* < 0.001). No significant difference was observed in levels of triglycerides (TG) and high density lipoprotein cholesterol (HDL-C) in the two groups (Table 1).

CIMT in the cases positively correlated with age (*r* = +0.357; *P* < 0.05), duration of disease (*r* = +0.549; *P* < 0.01), and BASMI (*r* = +0.337; *P* < 0.05). CIMT negatively correlated with cervical rotation (CR) (*r* = −0.296; *P* < 0.05), lateral flexion (LF) (*r* = −0.344; *P* < 0.05), intermalleolar distance (IMD) (*r* = −0.268; *P* < 0.05), lumbar flexion (*r* = −0.313; *P* < 0.05), and ESR (*r* = −0.295; *P* < 0.05). No significant correlation was observed between CIMT and age at onset of disease, BASDAI, BASFI, tragus-to-wall (TTW) distance, CRP, and HLA B-27 positivity. No significant correlation was observed between CIMT and HDL-C, LDL-C, and TG levels (Table 2).

## 4. Discussion

The threat of increased cardiovascular morbidity and mortality in AS and other rheumatic diseases, coupled with the possibility of CIMT measurements to reliably, noninvasively, and inexpensively screen it at a relatively nascent stage, prompted us to go for this study.

Today there is widespread acceptance of CIMT as a reliable and easily reproducible marker of preclinical atherosclerosis and future CVD risk [10].

Tyrrell et al. did a systematic review and meta-analysis of 60 such studies encompassing the entire spectrum of rheumatic diseases and observed a significantly increased CIMT in these patients as compared to matched healthy individuals [11].

In our study, we observed a significant increase in CIMT in patients of AS (0.62 ± 0.12 mm) as compared to the matched group (0.54 ± 0.04 mm) (*P* < 0.001). These findings echo the observations of other authors all of whom had observed similar increases in CIMT [12–18].

Other studies also observed an increase of CIMT in AS cases as against the comparison group, but it was not statistically significant [19–21]. Nonetheless, a meta-analysis by Mathieu et al., studying 6 studies on CIMT in AS, concluded that there was a significant increase in CIMT in AS when compared with matched controls [22].

The findings of our study assume added significance because we excluded the other major risk factors of atherosclerosis, namely, diabetes mellitus, hypertension, chronic kidney disease, past or family history of premature myocardial infarction, and smoking, from our study and control group. Also we observed a statistically significant difference in CIMT while studying a much younger population (mean age 29.43 ± 9.00 years), as compared to Cece et al. [12] (36.8 ± 9.8), Hamdi et al. [13] (36 ± 11), Skare et al. [15] (42.89 ± 11.70), Peters et al. [17] (39), and Mathieu et al. [22] (40).

Choe et al. [20] had a study group with age comparable to ours (31.8 ± 6.8), but they did not observe a significant difference. Cardiovascular affection in inflammatory arthritis has been very commonly observed because of chronic inflammation being a common thread between rheumatology and preventive cardiology [7]. In light of the evidence before us it will be prudent to further evaluate CIMT in larger studies, as a small sample size remains a drawback of all these studies including ours.

In our study LDL-C was significantly lower in cases (*P* < 0.05). TG was observed to be lower and HDL-C higher in the study group, but this was not statistically significant (*P* > 0.05). Mathieu et al. [22], in a systematic review and meta-analysis of CVD risk in AS, noted a decrease in TG, LDL-C, and HDL-C levels. Divecha et al. [23] also observed lower levels of total cholesterol and HDL-C in AS. Malesci et al. [19] had observed LDL-C to be significantly higher (*P* = 0.03) and HDL-C significantly lower (*P* < 0.001) in patients of AS when compared to controls. The paradox of a seemingly beneficial lipid profile predisposing to accelerated atherosclerosis is seen in rheumatoid arthritis (RA) as well. The mechanism behind it is the ability of chronic inflammation to induce changes in the structural composition of the lipid molecules, making them more atherogenic [24]. Chronic inflammation also has the ability to induce endothelial cell dysfunction, wherein there is increased expression of leukocyte adhesion and signalling molecules on their surface. These changes are brought about by a complex interplay of inflammatory cytokines such as IL-6 and TNF-*α*, lipid molecules, and the endothelial cells [25].

In our study we also analyse the correlation of CIMT with markers of disease chronicity and activity in AS. CIMT was found to be positively correlated with age of the patient. We observed CIMT to increase with higher age at onset of disease, but the correlation was not statistically significant. In contrast, a strong correlation between these two parameters was observed in one previous study [13] (*P* = 0.001). We observed that CIMT positively correlated with disease duration (Table 2); in contrast some authors did not observe any significant correlation of CIMT and disease duration [12, 15–17].

CIMT positively correlated with decreased spinal mobility, measured using BASMI scores, consistent with the findings of other authors [13, 16, 21] while being in variance to some authors [12, 17].

In our study no correlation was observed between CIMT and BASDAI and BASFI scores, which concurs with some studies [15, 17, 21] and differs from some studies [13, 16, 20].

BASDAI and BASFI scores are the most extensively used scoring systems to make a clinical assessment of disease activity. But it must be pointed out that they suffer from an element of subjectivity as they take into account only the patient's perception of his/her disease burden. Also, BASDAI and BASFI scores indicate the inflammatory activity at the time of assessment. They do not tell us about the inflammatory burden of the entire disease duration. On the other hand, the metrological measurements provide us with an objective assessment of the degree of damage that the axial skeleton has to suffer during the entire disease process. Hence, they can be thought of as surrogate markers of disease chronicity. The correlation of disease duration and metrological measurements with CIMT can be explained by the fact that it is chronic systemic inflammation which is driving atherosclerosis in these patients.

No correlation was observed between CIMT and TG, HDL-C, and LDL-C. Similarly, no correlation was observed between CIMT and CRP levels in patients of AS. This can be explained by the fact that unlike RA, CRP is not a good marker of disease activity in AS. It is positive only in 50–60% of cases of AS and correlates poorly with disease activity [26]. Skare et al. [15] and Peters et al. [17] had also made similar observations.

A negative correlation was observed between CIMT and ESR (*r* = −0.295; *P* < 0.05). A plausible explanation for the same might be that ESR levels are an indicator of the present level of disease activity and do not show the inflammatory burden which has built up over time. This finding, otherwise, cannot be fully explained. A positive correlation between CIMT and ESR was observed in only one study [13].

Out of the 37 cases studied, 28 (75.68%) were positive for HLA B-27 and 9 (24.32%) were negative. Increase in CIMT was not found to be associated with HLA B-27 positivity in our study, the same as in other studies [13, 15].

## 5. Conclusion

Increased CIMT was observed in the present study in patients of AS as compared to normal subjects and it correlated with the BASMI, an index of chronicity of disease.

## Figures and Tables

**Table 1:** Characteristics of study subjects.

	Cases of AS (37)	Healthy controls (37)	*P*
Age (range) (years)	29.43 ± 9.0 (17–50)	29.43 ± 9.0 (17–50)	>0.05
Age at onset (years)	24.01 ± 8.36	—	
Disease duration (months)	65.62 ± 54.92	—	
BASDAI	4.11 ± 1.99	—	
BASFI	4.13 ± 2.09	—	
BASMI	3.14 ± 2.14	—	
ESR (mm/hr)	45.19 ± 26.04	—	
CRP (mg/dL)	7.36 ± 3.78	—	
HLA-B27 (% of cases)	75.68		
CIMT (mm)	0.62 ± 0.12	0.54 ± 0.04	<0.001
TG (mg/dL)	93.65 ± 36.18	108.62 ± 41.52	>0.05
HDL-c (mg/dL)	47.92 ± 6.44	44.92 ± 3.86	>0.05
LDL-c (mg/dL)	80.96 ± 21.66	107.36 ± 25.84	<0.001

**Table 2:** Correlation of CIMT with markers of disease severity and chronicity.

	Correlation coefficient (*r*)	*P*
CIMT and age	+0.357	**<0.05**
CIMT and disease duration	+0.549	**<0.01**
CIMT and age at onset	+0.086	>0.05
CIMT and BASDAI	−0.160	>0.05
CIMT and BASFI	+0.059	>0.05
CIMT and TTW	+0.098	>0.05
CIMT and lateral flexion	−0.344	**<0.05**
CIMT and IMD	−0.268	**<0.05**
CIMT and CR	−0.296	**<0.05**
CIMT and lumbar flexion	−0.313	**<0.05**
CIMT and BASMI	+0.337	**<0.05**
CIMT and ESR	−0.295	**<0.05**
CIMT and CRP	−0.133	>0.05
CIMT and TG	−0.024	>0.05
CIMT and HDLc	+0.106	>0.05
CIMT and LDLc	+0.189	>0.05
CIMT and HLA B-27 positivity	—	>0.05

## References

[1] Bruce IN, Gladman DD, Urowitz MB (2000). Premature atherosclerosis in systemic lupus erythematosus. *Rheumatic Disease Clinics of North America*.

[2] Gabriel SE (2008). Cardiovascular morbidity and mortality in rheumatoid arthritis. *American Journal of Medicine*.

[3] Crowley JJ, Donnelly SM, Tobin M (1993). Doppler echocardiographic evidence of left ventricular diastolic dysfunction in ankylosing spondylitis. *American Journal of Cardiology*.

[4] Yildirir A, Aksoyek S, Calguneri M, Oto A, Kes S (2002). Echocardiographic evidence of cardiac involvement in ankylosing spondylitis. *Clinical Rheumatology*.

[5] Szabo SM, Levy AR, Rao SR (2011). Increased risk of cardiovascular and cerebrovascular diseases in individuals with ankylosing spondylitis: a population-based study. *Arthritis and Rheumatism*.

[6] Peters MJ, Van Der Horst-Bruinsma IE, Dijkmans BA, Nurmohamed MT (2004). Cardiovascular risk profile of patients with spondylarthropathies, particularly ankylosing spondylitis and psoriatic arthritis. *Seminars in Arthritis and Rheumatism*.

[7] Libby P, Ridker PM, Maseri A (2002). Inflammation and atherosclerosis. *Circulation*.

[8] Van den Oever IAM, van Sijl AM, Nurmohamed MT (2013). Management of cardiovascular risk in patients with rheumatoid arthritis: evidence and expert opinion. *Therapeutic Advances in Musculoskeletal Disease*.

[9] Greenland P, Alpert JS, Beller GA (2010). 2010 ACCF/AHA guideline for assessment of cardiovascular risk in asymptomatic adults: a report of the American College of Cardiology Foundation/American Heart Association Task Force on Practice Guidelines. *Journal of the American College of Cardiology*.

[10] O’Leary DH, Polak JF, Kronmal RA, Manolio TA, Burke GL, Wolfson SK (1999). Carotid-artery intima and media thickness as a risk factor for myocardial infarction and stroke in older adults. *The New England Journal of Medicine*.

[11] Tyrrell PN, Beyene J, Feldman BM, McCrindle BW, Silverman ED, Bradley TJ (2010). Rheumatic disease and carotid intima-media thickness: a systematic review and meta-analysis. *Arteriosclerosis, Thrombosis, and Vascular Biology*.

[12] Cece H, Yazgan P, Karakas E (2011). Carotid intima-media thickness and paraoxonase activity in patients with ankylosing spondylitis. *Clinical and Investigative Medicine*.

[13] Hamdi W, Bouaziz MC, Zouch I (2012). Assessment of preclinical atherosclerosis in patients with ankylosing spondylitis. *Journal of Rheumatology*.

[14] Mathieu S, Joly H, Baron G (2008). Trend towards increased arterial stiffness or intima-media thickness in ankylosing spondylitis patients without clinically evident cardiovascular disease. *Rheumatology*.

[15] Skare TL, Verceze GC, de Oliveira AA, Perreto S (2013). Carotid intima-media thickness in spondyloarthritis patients. *Sao Paulo Medical Journal*.

[16] Bodnár N, Kerekes G, Seres I (2011). Assessment of subclinical vascular disease associated with ankylosing spondylitis. *The Journal of Rheumatology*.

[17] Peters MJL, Van Eijk IC, Smulders YM (2010). Signs of accelerated preclinical atherosclerosis in patients with ankylosing spondylitis. *Journal of Rheumatology*.

[18] Gonzalez-Juanatey C, Vazquez-Rodriguez TR, Miranda-Filloy JA (2009). The high prevalence of subclinical atherosclerosis in patients with ankylosing spondylitis without clinically evident cardiovascular disease. *Medicine*.

[19] Malesci D, Niglio A, Mennillo GA, Buono R, Valentini G, La Montagna G (2007). High prevalence of metabolic syndrome in patients with ankylosing spondylitis. *Clinical Rheumatology*.

[20] Choe J-Y, Lee M-Y, Rheem I, Rhee M-Y, Park S-H, Kim S-K (2008). No differences of carotid intima-media thickness between young patients with ankylosing spondylitis and healthy controls. *Joint Bone Spine*.

[21] Sari I, Okan T, Akar S (2006). Impaired endothelial function in patients with ankylosing spondylitis. *Rheumatology*.

[22] Mathieu S, Gossec L, Dougados M, Soubrier M (2011). Cardiovascular profile in ankylosing spondylitis: a systematic review and meta-analysis. *Arthritis Care & Research*.

[23] Divecha H, Sattar N, Rumley A, Cherry L, Lowe GDO, Sturrock R (2005). Cardiovascular risk parameters in men with ankylosing spondylitis in comparison with non-inflammatory control subjects: relevance of systemic inflammation. *Clinical Science*.

[24] Haddy N, Sass C, Droesch S (2003). IL-6, TNF-*α* and atherosclerosis risk indicators in a healthy family population: The STANISLAS cohort. *Atherosclerosis*.

[25] Khovidhunkit W, Kim M-S, Memon RA (2004). Effects of infection and inflammation on lipid and lipoprotein metabolism: mechanisms and consequences to the host. *Journal of Lipid Research*.

[26] Van Der Horst-Bruinsma IE, Lems WF, Dijkmans BAC (2009). A systematic comparison of rheumatoid arthritris and ankylosing spondylitis. *Clinical and Experimental Rheumatology*.

